# Fecal carriage of extended spectrum beta-lactamase producing Enterobacteriaceae among HIV infected children at the University of Gondar Comprehensive Specialized Hospital Gondar, Ethiopia

**DOI:** 10.1186/s12981-021-00347-x

**Published:** 2021-04-21

**Authors:** Biruk Bayleyegn, Roman Fisaha, Desie Kasew

**Affiliations:** 1 Department of Clinical Hematology and Immunohematology, College of Medicine and Health Sciences, University of Gondar, 196 Gondar, Ethiopia; 2 Department of Medical Microbiology, College of Medicine and Health Sciences, University of Gondar, Gondar, Ethiopia

**Keywords:** Children, ESBL, Enterobacteriaceae, HIV

## Abstract

**Background:**

Human immunodeficiency virus (HIV) and extended spectrum beta lactamase (ESBL) producing Enterobacteriaceae infections are the major challenges in sub-Saharan Africa. Data on the carriage rate of ESBL producing Enterobacteriaceae among HIV infected children is lacking in Ethiopia. Hence this study was aimed to investigate fecal carriage of ESBL producing Enterobacteriaceae among HIV infected children at the University of Gondar comprehensive Specialized Hospital.

**Methods:**

A cross-sectional study was conducted among HIV infected children from January to April 2020. Stool specimens were collected from 161 study participants by convenient sampling and cultured on MacConkey agar. Biochemical identification, antimicrobial susceptibility testing including ESBL production were carried out. Data were analyzed by SPSS version-20 and P-value < 0.05 on multivariate logistic regression analysis was regarded as statistically significant.

**Results:**

From a total of 161 study participants male to female ratio was 1:1.1. Moreover; 96.3% of participants were in HIV stage-I and 90.1% had at least a year highly active antiretroviral therapy exposure. A total of 186 Enterobacteriaceae, with *E. coli* 60% and *K. pneumonia* 16.13% predominance were isolated from 161 participants. Majority of isolates were most resistant to amoxicillin (95.1%) and sensitive to CHL (94.1%), CXT (91.4%) and CAZ (91.4%). There were 71(38.17%) multidrug resistant isolates, 13 of which were also ESBL producers. The overall ESBL carriage rate was 32/161 (19.9%). History of antibiotic use was the independent factor associated with ESBL carriage (AOR 3.23 (95% CI 1.054–9.88)) and P-value of 0.04.

**Conclusion:**

ESBL carriage rate of HIV infected children was considerable. Previous antibiotic use was the independent factor. Regular screening for antibiotic resistance on HIV patients before prescription and large-scale antibiotic resistance survey including healthy community may be important.

## Introduction

Human immunodeficiency virus/Acquired immune deficiency syndrome (HIV/AIDS) infection which damages mucous membrane of intestine, baring the host to antimicrobial resistant infections [1]. Antimicrobial resistance including ESBL production is of significant impact globally mainly in the developing countries [2]. Moreover; the burden of ESBL among healthy people is increasing worldwide with an estimated of 5% rise per year [3]. More than two folds increased prevalence of ESBL producing Enterobacteriaceae in French children between 2010 and 2015 has grown from 4.8 to 10.2% [4]. In addition, a review by Lewis et al. in sub Saharan Africa reported a pooled estimate of ESBL carriage as 18%, indicating the highest burden in the region than reports from US and Europe (3.4 to 7.3%) [5].

ESBL enzymes confer resistance to penicillin, cephalosporin and monobactam [6]. Even some reports indicate the presence of carbapenem resistance [7]. Bacteria produce β-lactamases, a potent family of enzymes that breakdown β-lactam ring of antibiotics making it ineffective and become a major risk in an increasing burden of resistant infections [6]. Multidrug resistance (MDR) was defined as resistance to a minimum of one drug in three classes of antimicrobial agents [8].

East and South Africa region is the largest HIV burden area of the world. Ethiopia, as part of this region contributed to an estimated of 722,248 HIV carriers 2017 [9] and 62,000 children living with HIV/AIDS were reported in 2016 [10]. The immune status of HIV patients is weakened and are at an increased risk of infections, hospitalization and antibiotic consumption than HIV free individuals [11]. Health care associated infection, mechanical ventilation, use of invasive medical devices, prolonged hospital stay and antibiotic use are among factors contributing to increased burden of ESBL producing Enterobacteriaceae [6, 7].

Intestinal carriage of ESBL producing Enterobacteriaceae can cause infections with increased hospital stay, loss of treatment options and associated healthcare costs even leads to death [12]. Hence, the problem needs an emphasis, investigation of ESBL carriage is considered as one of the targets that help to fight antimicrobial resistant infections particularly in resource constrained settings [13]. Data on the carriage rate of ESBL producing Enterobacteriaceae in the general population is very limited in Ethiopia. In spite of their high risk to multiple infections and resulting exposure several antibiotics, the problem among children living with the HIV is overlooked in the country. Hence, this study was aimed to investigate the intestinal carriage rate of ESBL among HIV infected children at the University of Gondar comprehensive Specialized Hospital.

## Methods

### Study area and population

Institutional cross-sectional study was conducted at the University of Gondar comprehensive Specialized Referral Hospital (UoGCSH) antiretroviral therapy (ART) clinic from January to April 2020. The hospital is found in Gondar town, Amhara region Ethiopia. The town is found at 740 km in the northwest of the capital city, Addis Ababa. The University of Gondar Specialized Referral Hospital is the teaching hospital which provides teaching activities to medical and health science students and the oldest academic institution in Ethiopia. It provides medical, surgical, psychiatric, and many other services to more than 7 million people of the Gondar province and the neighboring regions. The hospital has both inpatient with more than 512 beds and outpatient services. It also provides HIV/AIDS intervention activities like free diagnosis, treatment and monitoring in its ART clinic.

### Population

All HIV-infected children who were attending at UoGSRH ART clinic were the source population while, all HIV infected children who visit UOGSRH ART clinic during the study period were our study population. All HIV infected children who are under 15 years old were included in the study while children without legal guardian or unaccompanied children were excluded from the study.

### Socio-demographic and clinical data collection

A pretested questionnaire was employed to collect socio-demographic data of the study participants including age, gender, residence, educational status, family size, family income, family occupation and food habit by face to face interview. In addition, clinical data of the study participants such as, history of invasive medications, World Health Organization (WHO) disease stage of HIV, opportunistic infections, presence of fever and diarrhea, HAART experience and type of HAART, duration of HAART and recent history of antibiotic use were collected by reviewing the medical record of HIV infected children. Anthropometric measurements including weight and height were measured by digital scale. The data collection was investigator administered on site to all participants and/or their guardians at the ART follow up clinic of UoGCSH.

### Laboratory procedures

Fresh stool specimen was collected using a codded clean leak proof plastic cup and transported to Medical microbiology laboratory for culture within 2 h of collection. The collected stool specimen was inoculated on MacConkey agar medium and then incubated at 37℃ for 24 h for selective growth of Gram-negative bacteria and lactose fermentation characteristics. Biochemical tests were performed for species identification. Once the species of Enterobacteriaceae were identified, antimicrobial susceptibility test (AST) was performed using Kirby-Bauer Disk Diffusion susceptibility test method on Mueller Hinton agar (MHA). Antimicrobial agents such as ampicillin, amoxicillin-clavulanate, Trimethoprim-Sulfamethoxazole, chloramphenicol, cefixime, cefoxitin, ceftazidime, cefotaxime, tetracycline and ciprofloxacin were used for AST. Discs were selected based on their availability in the local treatment following CLSI-2019. Isolates were also screened for ESBL enzyme production by applying Cefotaxime 30 µg and Ceftazidime 30 µg discs. Phenotypic confirmation of ESBL production was performed using combination disc diffusion method. After screening, simultaneous application of Cefotaxime 30 µg and Ceftazidime 30 µg with their respective combination with clavulanate 10 µg (Cefotaxime–clavulanate 30–10 µg, Ceftazidime–clavulanate 30–10 µg) was used for confirmation following the guideline of Clinical Laboratory Standards Institute (CLSI-2019). Susceptibility was done by preparing suspension of pure isolates comparable to 0.5Mcfurland standard. The difference in their zone of inhibition were measured after inoculation on MHA and incubation aerobically at 37˚C for 16-18hrs. A change in diameter zone of 5 mm and above for either cefotaxime or ceftazidime or both from their combined form was reported as ESBL producing isolate. *Klebsiella pneumoniae* ATCC 700,603 (positive control) and *Escherichia coli* strain ATCC 25,922 (negative control) were used for quality control (CLSI-2019) [14].

### Data analysis

Data were entered to epi-data version 4.1 and exported to statistical packages for social sciences (SPSS) version-20 for analysis. The results were presented in frequency and percentage through table and text. Univariate and multivariate logistic regression were used to assess the association between the independent variables and the occurrence of ESBL producing Enterobacteriaceae. A variable with *P*-value of < 0.05 was considered as statistically significant.

## Results

### Sociodemographic variables of the study population

A total of 161 study participants were recruited in the study with male to female ratio of 1:1.09. Majority of participants were above 10 years (77%), urban residents (87%) and in the WHO HIV stage I (96.3%). Moreover; 46.6% of participants had stunted growth, 34.8% had viral load of > 1000 copies/ml and 90.1% had been taking HAART for at least a year (Table 1).

**Table 1:** The Sociodemographic and clinical characteristics of HIV infected children at the University of Gondar Comprehensive Specialized Hospital, 2020

Variable	Category	Frequency (N)	Percent (%)
Gender of children	Male	77	47.8
	Female	84	52.2
Age (years) of children	Less than or equal to10	37	23.0
	11 and above	124	77.0
Residence	Urban	140	87.0
	Rural	21	13.0
Educational status of children	No formal education	12	7.5
	Primary school	130	80.7
	Secondary school	19	11.8
Family income per month	Less than 1000	77	47.8
	1000–2000	54	33.5
	2000 birr and above	30	18.6
Family size	Less than or equal to 4	113	70.2
	5 and above	48	29.2
Family occupation	Privately employed	15	9.3
	Government worker	43	26.7
	Merchant	49	30.4
	House wife	43	26.7
	Other*	11	6.8
Family educational status	No formal education	55	34.2
	Primary school	49	30.4
	Secondary and above	57	35.4
HIV status of caregiver	Positive	134	83.2
	Negative	17	10.6
	Not known	10	6.2
WHO stage of HIV	I	155	96.3
	II and late stage	6	3.7
WAZ	Under weight	11	6.8
	Normal	150	93.2
HAZ	Stunted	75	46.6
	Normal	86	53.4
BAZ	Wasted	30	18.6
	Normal	131	81.4
Opportunistic infections	Yes	23	14.3
	No	138	85.7
Presence of fever	Yes	20	12.4
	No	141	87.6
History of antibiotic use	Yes	15	9.3
	No	146	90.7
Presence of diarrhea	Yes	13	8.1
	No	148	91.9
Viral load	Not detected	61	37.9
	Less than or equal to 1000 copies /ml	44	27.3
	Greater than 1000 copies/ml	56	34.8
HAART experience	Less than 6 months	9	5.6
	6–12 months	7	4.3
	12 months and above	145	90.1
HAART type	AZT based	55	34.1
	TDF based	61	37.9
	ABC based	45	28.0
Eating uncooked products	Yes	110	68.3
	No	51	31.7
Eating row vegetable	Yes	114	70.8
	No	47	29.2
Cytopenia	Cytopenia	54	33.5
	Normal	102	63.4

Other*: farmers, daily manual workers, those without work

WAZ: Weight-for-age; HAZ: Height-for-age; BAZ: Weight-for-Height

### Distribution and antimicrobial resistance profile of Enterobacteriaceae

Among 161 study participants who brought stool specimen, there were 186 Enterobacteriaceae isolated from stool culture. *E. coli* was the most common isolate 59.7% followed by *K. pneumoniae* 16.13%. All of isolates were highly resistant to Amoxicillin (95.1%) and Ampicillin (85%). Specifically, more than 96% of *E. coli* and *K. pneumoniae* were resistant to Amoxicillin while, 85.6% *E. coli* and 90% *K. pneumoniae* were resistant to Ampicillin. Similarly, 47.7% of *E. coli* and 46.7% of *K. pneumoniae* were resistant tetracycline. On the other hand, 90%, 92.8%, 97.3% of *E. coli* were sensitive to CAZ, CHL and CXT respectively. High proportion of *K. pneumoniae* were resistant to CXM (90%), CAZ (93.3%) and CHL (93.3%) (Table 2).

**Table 2:** Antimicrobial Susceptibility pattern of Enterobacteriaceae isolates among HIV infected children at the University of Gondar Comprehensive Specialized Hospital, 2020

Name of antibiotics		Antimicrobial resistance pattern of each species of *Enterobacteriacae*									
		*E. coli* (N = 111)	*K.pneumoniae* (N = 30)	*K.ozenia* (N = 4)	*Proteus* species (N = 4)	*Providencia* species (N = 3)	*Citrobacter* species (N = 9)	*Eenterobacter* species (N = 12)	*Salmonella* species (N = 7)	*Shigella* species (N = 6)	Row total N (%)
AMP	S	3 (2.7)	1 (3.3)	2 (50)				2 (16.7)			8 (4.3)
	I	13 (11.7)	2 (6.7)				2 (22.2)			3 (50)	20 (10.8)
	R	95 (85.6)	27 (90)	2 (50)	4 (100)	3 (100)	7 (77.8)	10 (83.3)	7 (100)	3 (50)	158 (85)
AMX	S	1 (0.9)	1 (3.3)	2 (50)			1 (11.1)	1 (8.3)			6 (3.2)
	I	3 (2.7)									3 (1.6)
	R	107 (96.4)	29 (96.7)	2 (50)	4 (100)	3 (100)	8 (88.9)	11 (91.7)	7 (100)	6 (100)	177 (95.1)
AMC	S	41 (36.9)	11 (36.7)	2 (50)	1 (25)	2 (66.7)	2 (22.2)	5 (41.7)	2 (28.6)	3 (50)	68 (36.6)
	I	17 (15.3)	8 (26.7)	1 (25)			2 (22.2)		1 (14.3)	1 (16.7)	30 (16.1)
	R	53 (47.7)	11 (36.7)	1 (25)	3 (75)	1 (33.3)	5 (55.6)	7 (58.3)	4 (57.1)	2 (33.3)	87 (46.8)
CTX	S	97 (87.4)	26 (86.7)	4 (100)	4 (100)	2 (66.7)	8 (88.9)	8 (66.7)	6 (85.7)	6 (100)	161 (86.6)
	I	3 (2.7)				1 (33.3)		1 (8.3)			5 (2.7)
	R	11 (9.9)	4 (13.3)				1 (11.1)	3 (25)	1 (14.3)		20 (10.8)
CAZ	S	100 (90)	28 (93.3)	4 (100)	4 (100)	3 (100)	8 (88.9)	12 (100)	6 (85.7)	6 (100)	(91.9)
	I	2 (1.8)									2 (1.1)
	R	9 (8.1)	2 (6.7)				1 (11.1)		1 (14.3)		13 (7)
CXM	S	99 (89.2)	27 (90)	4 (100)	4 (100)	3 (100)	6 (66.7)	9 (75)	7 (100)	6 (100)	165 (88.7)
	I	4 (3.6)									4 (2.2)
	R	8 (7.2)	3 (10)				3 (33.3)	3 (25)			17 (9.1)
CXT	S	108 (97.3)	25 (83.3)	4 (100)	3 (75)	3 (100)	6 (66.7)	10 (83.3)	7 (100)	5 (83.3)	171 (91.9)
	I	1 (0.9)	2 (6.7)					1 (8.3)		1 (16.7)	5 (2.7)
	R	2 (1.8)	3 (10)		1 (25)		3 (33.3)	1 (8.3)			10 (5.4)
CIP	S	82 (73.9)	22 (73.3)		2 (50)		7 (77.8)	11 (91.7)	5 (71.4)	4 (66.7)	133 (71.5)
	I	22 (19.8)	7 (23.3)	4 (100)	1 (25)	3 (100)	2 (22.2)		2 (28.6)	2 (33.3)	43 (23.1)
	R	7 (6.3)	1 (3.3)		1 (25)			1 (8.3)			10 (5.4)
SXT	S	63 (56.8)	17 (56.7)	2 (50)	1 (25)	1 (33.3)	4 (44.4)	7 (58.3)	1 (14.3)	4 (66.7)	100 (53.8)
	I	3 (2.7)		1 (25)				1 (8.3)			5 (2.7)
	R	45 (40.5)	13 (43.3)	1 (25)	3 (75)	2 (66.7)	5 (55.6)	4 (33.3)	6 (85.7)	2 (33.3)	81 (43.5)
CHL	S	103 (92.8)	28 (93.3)	4 (100)	4 (100)	3 (100)	8 (88.9)	12 (100)	7 (100)	6 (100)	175 (94.1)
	R	8 (7.2)	2 (1.8)				1 (11.1)				11 (5.9)
TET	S	52 (46.8)	15 (50)	3 (75)	1 (25)	3 (100)	6 (66.7)	7 (58.3)	2 (28.6)	2 (33.3)	88 (47.3)
	I	6 (5.4)	1 (3.3)							1 (16.7)	8 (4.3)
	R	53 (47.7)	14 (46.7)	1 (25)	3 (75)		3 (33.3)	5 (41.7)	5 (71.4)	3 (50)	87 (46.8)

AMP: ampicillin: AMX: amoxicillin; AMC: amoxicillin-clavulanic acid; CTX: cefotaxime; CAZ: ceftazidime; CXM: cefixime; CXT: cefoxitine; CIP: ciprofloxacin; SXT: Trimethoprim-Sulfamethoxazole; CHL: chloramphenicol; TET: tetracycline

### Distribution of MDR and ESBL producing Enterobacteriaceae

The proportion of MDR from the total Enterobacteriaceae isolates was 71/186 (38.7%) in this study. Of the MDR isolates, 46/71 (64.8%) were *E. coli* followed by 8/71 (11.3%) *K. pneumonia.* Moreover; thirteen of the MDR isolates (twelve *E. coli* and one *K. pneumonia*) were ESBL producers. Of the total (161) participants 19.9% were carriers of ESBL producing isolates. There was no double or multiple carriage of ESBL isolates. All ESBL producing isolates were *E. coli* (16.2%) and *K. pneumonia* (3.7%) (Table 3).

**Table 3:** Frequency of Enterobacteriaceae and distribution of MDR and ESBL producing isolates among HIV infected children at the University of Gondar Comprehensive Specialized Hospital, 2020

Species of isolate	Total No (%) of isolates	No of MDR isolates	% of MDR isolates, N (71)	%MDR from total isolates (186)	% ESBL from total sample size (32/161)	Both MDR and ESBL 13/186
*E. coli*	111 (59.7)	46	64.8	24.7	26 (16.2)	12 (6.5)
*K.pneumonia*	30 (16.1)	8	11.3	4.3	6 (3.7)	1 (0.5)
*K.ozenia*	4 (2.2)	1	1.4	0.5	–	–
*Proteus* species	4 (2.2)	3	4.2	1.6	–	–
*Citrobacter* species	9 (4.8)	4	5.6	2.2	–	–
*Enterobacter* species	12 (6.5)	2	2.8	1.1	–	–
*Salmonella* species	7 (3.8)	4	5.6	2.2	–	–
*Shigella* species	6 (3.2)	2	2.8	1.1	–	–
*Providencia* species	3 (1.6)	–	–	–	–	–
Total	186 (100)	71	100	38.7	19.9%	7%

### Factors associated with ESBL production

Among the factors analyzed in bivariate logistic regression, family education and history of antibiotic use had P-value of less than 0.2 and were fitted to multivariate analysis. But in multivariate analysis, only history of antibiotic use had statistically significant association with ESBL carriage (AOR, 3.23 95% CI 1.054–9.88) and P-value of 0.04 (Table 4).

**Table 4:** Factors associated with ESBL Production among HIV infected children at the University of Gondar Comprehensive Specialized Hospital, 2020

Variable	Category	ESBL carriage		COR (95% CI)	P-value	AOR (95% CI)	P-value
		Yes N (%)	N (%)				
Gender	Male	13 (16.9)	64 (83.1)	1			
	Female	18 (21.4)	66 (78.6)	1.34 (0.61–2.96)	0.47		
Age (years)	Less than or equal to10	7 (18.9)	30 (81.1)	1			
	11–15	24 (19.4)	100 (80.6)	1.03 (0.40–2.62)	0.95		
Residence	Urban	28 (20)	112 (80)	1			
	Rural	3 (14.3)	18 (85.7)	0.67 (0.18–2.42)	0.54		
Family income per month	Up to 1000 ETB	15 (19.5)	62 (80.5)	0.97 (0.34–2.79)	0.98		
	1000–2000 ETB	10 (18.5)	44 (81.5)	0.91 (0.29–2.81)	0.87		
	Above 2000 ETB	6 (20)	24 (80)	1			
Family size	Down to 4	21 (18.6)	92 (81.4)	1			
	5–8	10 (20.8)	38 (79.2)	1.15 (0.50–2.68)	0.74		
Family educational status	No	6 (10.9)	49 (89.1)	0.46 (0.16–1.33)	0.15	0.47 (0.15–1.30)	0.13
	Primary school	13 (26.5)	36 (73.5)	1.35 (0.55–3.33)	0.51	1.27 (0.5–3.17)	0.61
	Secondary school & above	12 (21.1)	45 (78.9)	1		1	
WHO stage	I	29 (18.7)	126 (81.3)	1			
	II	2 (33.3)	4 (66.7)	2.17 (0.38–12.44)	0.38		
BAZ	Underweight	8 (26.7)	22 (73.3)	1.71 (0.68–4.31)	0.26		
	Normal	23 (17.6)	108 (82.4)	1			
HAZ	Stunted	12 (16)	63 (84)	1.45 (0.67–3.32)	0.33		
	Normal	19 (22.1)	67 (77.9)	1			
Presence of fever	Yes	4 (20)	16 (80)	1.06 (0.33–3.41)	0.93		
	No	27 (19.1)	114 (80.9)	1			
Opportunistic infections	Yes	6 (26.1)	17 (73.9)	1.60 (0.57–4.45)	0.37		
	No	25 (18.1)	113 (81.9)	1			
Diarrhea	Yes	3 (23.1)	10 (76.9)	1.29 (0.33–4.98)	0.72		
	No	28 (18.9)	120 (81.1)	1			
History of antibiotic use	Yes	6 (40)	9 (60)	3.23 (1.05–9.88)	**0**.**04**	3.2 (1.05–9.9)	**0.04**
	No	25 (17.1)	121 (82.9)	1		1	
Eating animal products	Yes	24 (21.8)	86 (78.2)	1.75 (0.70–4.39)	0.23		
	No	7 (13.7)	44 (86.3)	1			
Eating green vegetables	Yes	23 (20.2)	91 (79.8)	1.23 (0.51–2.99)	0.65		
	No	8 (17)	39 (83)	1			
Cytopenia	Normal	21 (19.6)	86 (80.4)	1			
	Cytopenia	10 (18.5)	44 (81.5)	0.93 (0.40–2.14)	0.87		
Viral load	≤ 1000 copy /ml	6 (13.6)	16 (69.6)	0.53 (0.19–1.51)	0.24		
	> 1000 copy/ml	11 (19.6)	45 (80.4)	0.82 (0.34–2.00)	0.66		
	Not detected	14 (23)	47 (77)	1			
HAART type	AZT based	10 (18.2)	45 (81.8)	0.78 (0.29–2.08)	0.62		
	TDF based	11 (18)	50 (82)	0.77 (0.29–2.01)	0.59		
	ABC based	10 (22.2)	35 (77.8)	1			

Bold numeral represented statistically significant

## Discussion

The overall carriage rate of ESBL producing Enterobacteriaceae among children living with HIV was 19.9% (95% CI 14.4–26.1). The result was comparable to reports among healthy children (16%) [15] and (22%) [16] in Cameroon**,** 21% Madagascar [17], (24.8%) Lebanon [18] and 16.8% Sweden [19]. On the other hand, the result was lower compared to reported results 31% in Niger [20], 34.3% Tanzania [21], 59% Central Africa Republic [22], 32.6% Guinea Bissau [23] and 49.6% Lebanon [24]. The fecal carriage of ESBL producing Enterobacteriaceae in this study was a little higher than 13.7% in Zimbabwe [25] and higher than reports among HIV negative children from different geographic regions: (10%) in Kenya [26], (5.0%) Ghana [27], (3.5%) United States of America [28], (4.6%) France [29], (4.7%) South Africa [12]. Geographic variation, variation in the method of ESBL detection or measures taken for the prevention of antimicrobial resistance might be responsible for the difference.

The rate of antimicrobial resistance in this study were highest against Amoxicillin (95.1%), Ampicillin (85%), Amoxicillin-clavulanic acid (46.8%), Tetracycline (46.8%) and Trimethoprim-sulfamethoxazole (43.5%). However; lowest resistance to ceftazidime (91.9), cefoxitin (91.9%) and chloramphenicol (94.1%) was observed. The result concords to a report from Arba Minch, Ethiopia [30]. This high proportion of resistance indicates isolates could probably adapt themselves to the commonly prescribed antibiotics. Trimethoprim-sulphamethoxazole is a prophylactically prescribed to HIV infected children in Ethiopia as per WHO recommendations to resource limited settings [31]. Comparable resistance to trimethoprim-sulphamethoxazole was reported in Nepal 48.9% [32], Nigeria 54% [33]. But resistance rate found in this study was lower than 91.3% in Madagascar [17]. The difference might be due to difference in population.

The overall MDR rate of Enterobacteriaceae isolates among HIV infected children were 71/186 (38.2%). The two most common MDR isolates were *E. coli* 46/71 (64.8%) and *K. pneumonia* 8/71 (11.3%). In addition, 13/71 (18.3%) MDR isolates (twelve *E. coli* and one *K. pneumonia*) were ESBL producers. This result was lower than 68.3% MDR in Addis Ababa, Ethiopia [34]. This may be due to the difference in the study population and this ESBL producing Enterobacteriaceae may transfer their resistant trait to the naïve enteric commensals.

Among the total 161 HIV infected children included in this study, 32(19.9%) were carriers of phenotypically confirmed ESBL producing Enterobacteriaceae. The result was higher than 5.3% prevalence in Uganda [35] but lower than 28.46% in Nepal [32]. In this study there was no double or multiple carriage of ESBL isolates and all of the ESBL producing isolates were *E. coli* 26 (16.2%) and *K. pneumonia* 6 (3.7%). This high resistance to multiple antimicrobial agents in addition to ESBL production is a bottle neck in the treatment of infectious diseases and pushes to the utilization of last resort drugs resulting in loss of effective treatment option [36]. Over use, frequent and intermittent use together with ease of access to the antibiotics without prescription from private pharmacies could be the possible rationale for the increased resistance.

The history of antibiotic use has shown statistically significant association. The ESBL carriage rate among children with drug use was more than 3 times compared to their counter parts (AOR 3.2, 95% CI 1.05–9.9). History of antibiotic use is also reported as a risk factor by several studies too [15, 21]. A study also has reported high family income as an independent factor associated with increased risk of ESBL carriage. Because low family income limits the rate of exposure to antibiotics which intern reduces the risk of antibiotic resistance and ESBL carriage [22]. On the other hand, low family income was reported as significant factor associated with increased risk of ESBL carriage [21]. The result of this study in contrast showed that ESBL producing isolates were distributed irrespective of the income level and no significant association was found. Family size appeared to be associated with ESBL carriage in bivariate analysis but in multivariate analysis it was not significantly associated. Residence was reported as a factor by a study done among Hospitalized patients at Arba Minch, Ethiopia [30]. But it didn’t show statistically significant association with ESBL carriage in this study. In addition, in this study, age was not significantly associated with ESBL carriage which is in agreement with a report in Madagascar [17]. This study included only HIV infected children who had visited the hospital during the study period. Advanced techniques of ESBL detection were not used in this study.

## Conclusion

Fecal carriage of MDR and ESBL producing Enterobacteriaceae among HIV infected children was considerable. The History of antibiotic use was the independent factor associated with the carriage of ESBL producing Enterobacteriaceae. Regular screening of HIV patients for the carriage of ESBL producing isolates need to be strengthened. In addition, large scale antibiotic resistance survey including healthy community could be important.

## Data Availability

All the data are incorporated in the manuscript.

## Abbreviations

AOR: Adjusted odds ratio
ART: Antiretroviral therapy
AST: Antimicrobial susceptibility test
ATCC: American type culture collection
CLSI: Clinical Laboratory Standard Institute
ESBL: Extended spectrum beta lactamase
HAART: Highly active antiretroviral therapy
HIV/AIDS: Human immunodeficiency virus/Acquired immune deficiency syndrome
MDR: Multidrug resistance
MHA: Mueller Hinton Agar
UoGCSH: University of Gondar comprehensive Specialized Referral Hospital
WHO: World Health Organization
